# The Inducible microRNA-21 Negatively Modulates the Inflammatory Response in Teleost Fish via Targeting IRAK4

**DOI:** 10.3389/fimmu.2019.01623

**Published:** 2019-07-16

**Authors:** Qing Chu, Xiaolong Yan, Lihua Liu, Tianjun Xu

**Affiliations:** ^1^Laboratory of Fish Molecular Immunology, College of Fisheries and Life Science, Shanghai Ocean University, Shanghai, China; ^2^Laboratory of Marine Biology and Biotechnology, Qingdao National Laboratory for Marine Science and Technology, Qingdao, China; ^3^National Pathogen Collection Center for Aquatic Animals, Shanghai Ocean University, Shanghai, China; ^4^Key Laboratory of Exploration and Utilization of Aquatic Genetic Resources, Shanghai Ocean University, Ministry of Education, Shanghai, China; ^5^International Research Center for Marine Biosciences at Shanghai Ocean University, Ministry of Science and Technology, Shanghai, China

**Keywords:** miR-21, inflammation, IRAK4, NF-κB signaling, fish

## Abstract

Eradication of bacterial infection requires timely and appropriate immune and inflammatory responses, but excessive induction of inflammatory cytokines can cause acute or chronic inflammatory disorders. Thus, various layers of negative regulators and mechanisms are needed to ensure maintenance of the homeostasis for the immune system. miRNAs are a family of small non-coding RNAs that emerged as significant and versatile regulators involved in regulation of immune responses. Recently, the molecular mechanisms of miRNA in host-pathogen interaction networks have been extensively studied in mammals, whereas the underlying regulatory mechanisms in fish are still poorly understood. In this study, we identify miR-21 as a negative regulator of the teleost inflammatory response. We found that lipopolysaccharide and *Vibrio anguillarum* significantly upregulated the expression of fish miR-21. Upregulated miR-21 suppresses LPS-induced inflammatory cytokine expression by targeting IL-1 receptor-associated kinase 4 (IRAK4), thereby avoiding excessive inflammatory responses. Furthermore, we demonstrated that miR-21 regulates inflammatory responses through NF-κB signaling pathways. The collective findings indicate that miR-21 plays a regulatory role in host-pathogen interactions through IRAK4-mediated NF-κB signaling pathway.

## Introduction

MicroRNAs (miRNAs) are highly conserved, single-stranded, small non-coding RNAs that have been shown to act as essential regulators of gene expression at the posttranscriptional level. miRNAs induce gene degradation and suppression of translation, or both, mainly by imperfect binding to miRNA recognition elements (MREs) within the 3′-untranslated region (UTR) of target mRNAs (1). The specificity of miRNAs is thought to be mainly mediated by the residues 2–8 of the 5′end, also known as the seed region (2). By modulating gene expression, many miRNAs have been reported to regulate diverse biological processes, such as embryogenesis, tumorigenesis, differentiation, immunity, and inflammation (3, 4).

Innate immune and inflammatory responses are normal self-protection mechanism that eliminate pathogens and resist microbial invaders (5, 6). Microbial pathogen recognition relies on several classes of germline-encoded pattern recognition receptors (PRRs), including C-type lectin receptors, nucleotide oligomerization domain-like receptors (NLR), RIG-I-like receptors (RLRs), and toll-like receptors (TLRs) (6). TLRs participate in controlling multiple aspects of the innate immune response. Following recognition of TLR ligands, TLRs elicit innate immunity by activating multiple intracellular signaling cascades including variable adaptor proteins and transcriptional factors. Activation of NF-κB result in the transcription of various inflammatory genes, including TNF-α and IL-6, to sense microbial pathogens (7–9). Excessive immune and inflammatory responses can cause a wide spectrum of pathologies, thus resulting in either cell lesions or tissue damage. Thus, to maintain a balance between host preservation and inflammatory pathology, the immune response and inflammation need to be tightly regulated.

Growing evidence show that miRNAs play an important role in the regulation of immunity and inflammation. The influence of miRNAs on innate immune responses was first characterized in 2004 showing that miRNAs such as miR-142a, miR-181a, and miR-223 can control hematopoiesis (10). Since then, many miRNAs have been reported to be associated with immune responses (11, 12). For example, let-7i (13), let-7e (14), and miR-233 (15) can target TLR4, MyD88 has been shown to be regulated by miR-155 (16) and miR-149 (17), and TNF-α has been proven to be a target gene of miR-125b and miR-155 (18). These miRNAs exert pro- or anti-inflammatory effects at the immediate-early, early, and late response stages (12). Although the immune regulatory miRNA networks have been well-described in higher vertebrates, the regulation of these responses in fish remains largely unknown.

Miiuy croaker (*Miichthys miiuy*) belongs to the Sciaenidae family, which is distributed in the western North Pacific, including the Bohai Sea, the Yellow Sea, and the East Sea of China. They are economically important marine fish and processes important medicinal application value. The species has currently become an excellent model for studying fish immune responses due to extensively studying at its transcriptome, whole-genome, and functional gene and immune pathway regulation levels (19–22). As the complexity of fish living environment, they are constantly threatened by pathogenic microorganisms, of which Gram-negative bacteria are an important group. *Vibrio anguillarum* is a Gram-negative, commashaped rod bacterium, which can cause hemorrhagic septicemic disease affecting various fish and result in high mortality and enormous economic losses (23). Hence, the study on the regulatory mechanisms in response to *V. anguillarum* infection are particularly important and prominent, and it has always been the focus of our research.

In this study, we report the negative regulation role of miR-21 upon *V. anguillarum* infection. We found that miR-21 could be rapidly upregulated following the infection of *V. anguillarum*, as well as lipopolysaccharide (LPS) stimulation in miiuy croaker. Further analyses showed that upregulated miR-21 orchestrates the down-regulation of inflammatory cytokine genes through NF-κB signaling, and the inhibition is accomplished through the miR-21 target gene IL-1 receptor-associated kinase 4 (IRAK4). This tight negative regulation mechanism is critical for attenuating inflammatory response and avoiding excessive inflammation. These data not only provide the information on miRNA negative regulation in fish upon Gram-negative bacteria infection, but also enriched miRNA-mediated networks of host-pathogen interactions.

## Materials and Methods

### Animals Challenge

Miiuy croaker (~750 g) was obtained from Zhoushan Fisheries Research Institute, Zhejiang Province, China. Fish was acclimated in aerated seawater tanks at 25°C for several weeks before experiments. Bacterial challenge was performed as described previously (22, 24). Briefly, fish was challenged with 1 ml *V. anguillarum* (1.5 × 10^8^ CFU/ml) or 1 ml suspension of LPS (1 mg/ml) through intraperitoneal (24). As a comparison, 1 ml of physiological saline was used to challenge the individuals. Afterwards, fishes were respectively, sacrificed at different time point and the immune tissues (liver and spleen) were collected for RNA extraction. All animal experimental procedures were performed in accordance with the National Institutes of Health's Guide for the Care and Use of Laboratory Animals, and the experimental protocols were approved by the Research Ethics Committee of Shanghai Ocean University (No. SHOU-DW-2018-047).

### Cell Culture and Treatment

HEK293 cells were cultured in DMEM (Hyclone) medium supplemented with 10% Fetal Bovine Serum (FBS) (Gibco), 100 U/ml penicillin, and 100 μg/ml streptomycin at 37°C in 5% CO_2_. Miiuy croaker macrophages were isolated from head kidney samples as described (25). In brief, tissues were aseptically pushed through a 100-μm nylon mesh to give cell suspension which was then loaded onto 34%/51% Percoll (Pharmacia, USA) density gradient to obtain macrophages. The cells were cultured in L-15 (Hyclone) medium supplemented with 20% FBS and seeded into six-well plates at 26°C in 4% CO_2_ (24). For stimulation experiments, macrophages were challenged with ultrapure LPS and harvested at different times for RNA extraction. Cell viability was determined by trypan blue dye exclusion assay.

### Plasmids Construction

To construct the *IRAK4* 3′UTR reporter vector, the 3′UTR region of miiuy croaker *IRAK4* gene was amplified using PCR and cloned into pmir-GLO luciferase reporter vector (Promega) using *Sac I and Xba I* restriction sites. Similarly, the 3′UTR region of *Danio rerio IRAK4* gene (*Dr*IRAK4) was amplified and inserted into the *Sac I and Xba I* restriction sites of pmir-GLO vector. The mutant-type of *IRAK4* 3′UTR reporters were constructed by using Mut Express II Fast Mutagenesis Kit V2 (Vazyme) with specific primers (Table 1). Moreover, miiuy croaker *IRAK4* 3′UTR or its mutant-type reporters were inserted into the mVenus-C1 (Invitrogen) which included the sequence of enhanced green fluorescent protein (GFP). In addition, to construct the pre-miRNA vector, the pre-miR-21 sequence of miiuy croaker was PCR-amplified and then cloned into pcDNA3.1 vector (Invitrogen) (24). To construct the IRAK4 expression plasmid, the full length of CDS region and 3′UTR of miiuy croaker *IRAK4* gene were amplified by specific primer pairs with the Flag tag, and cloned into pcDNA3.1 vector (Invitrogen) (24). The correct construction of the plasmids was verified by Sanger sequencing and extracted through Endotoxin-Free Plasmid DNA Miniprep Kit (Tiangen).

**Table 1:** PCR primer information in this study.

**Primers**	**Sequences (5′-3′)**
**REAL-TIME PCR**	
miR-21-qRT-F	AGCAACAGCAGTCTGTAAG
miR-21-qRT-R	TCCAGTTTTTTTTTTTTTTTGCCA
5.8S rRNA-qRT-F	AACTCTTAGCGGTGGATCA
5.8S rRNA-qRT-R	GTTTTTTTTTTTTTTTGCCGAGTG
IRAK4-qRT-F	ATCGGCTAAGCGGACATCA
IRAK4-qRT-R	TACCTCGCCTCCATCAAGA
IL-1β-qRT-F	CATAAGGATGGGGACAACGAG
IL-1β-qRT-R	TAGGGGACGGACACAAGGGTA
IL-6-qRT-F	GCGGTAAAGGCATGGATAT
IL-6-qRT-R	GTTGTAGTTGGAAGGGCAG
IL-8-qRT-F	AGCAGCAGAGTCTTCGT
IL-8-qRT-R	TCTTCGCAGTGGGAGTT
β-actin-qRT-F	GTGATGAAGCCCAGAGCA
β-actin-qRT-R	CGACCAGAGGCATACAGG
**VECTOR CONSTRUCTION**	
pre-miR-21-F	CCCAAGCTTTGCGTCATGTCTTTGATAGT
pre-miR-21-R	CCGGAATTCGCATACAGACAGGTCGTTGT
IRAK4-3′UTR-Wt-SacI-F	CGCGAGCTCTTAAAGCACAGGGGTGAAG
IRAK4-3′UTR-Wt-XbaI-R	TGCTCTAGACTGCAGCAAATCCAGAGTT
IRAK4-3′UTR-Mt-F	AGTGTGCTCAGACGTCACAGTTTGATGGCTTAATATC
IRAK4-3′UTR-Mt-R	TGTGACGTCTGAGCACACTGCAGAGTAGTTGTAATACTT
*Dr* IRAK4-3′UTR-Wt-SacI-F	CGCGAGCTCAGGACACCTGGAGGAGCATC
*Dr* IRAK4-3′UTR-Wt-XbaI-R	TGCTCTAGACAGAGCGAGTCAAGCCGAAG
*Dr* IRAK4-3′UTR-Mt-F	GGAGCTGACTTTCGACTGGGGAACCTCAGACT
*Dr* IRAK4-3′UTR-Mt-R	AGTCGAAAGTCAGCTCCATGGTGGGACTCTTT
IRAK4-BamHI-F	CGCGGATCCAATAATTCAGTAACTTCCGC
IRAK4-XbaI-R	AACTCTGGATTTGCTGCAG
GFP-IRAK4-3′UTR-Wt-HindIII-F	CCCAAGCTTGCTAGTTAAAGCACAGGGGTGAAG
GFP-IRAK4-3′UTR-Wt-BamHI-R	CGCGGATCCCTGCAGCAAATCCAGAGTT
GFP-IRAK4-3′UTR-Mt-F	AGTGTGCTCAGACGTCACAGTTTGATGGCTTAATATC
GFP-IRAK4-3′UTR-Mt-R	TGTGACGTCTGAGCACACTGCAGAGTAGTTGTAATACTT

### miR-21 Target Identification

The miR-21 targets were predicted using Targetscan (2), miRanda (26), and MicroInspector (27) algorithms. Predictions were ranked based on the predicted efficacy of targeting as calculated using the context and scores of the sites.

### miRNA Mimics and Inhibitors

miR-21 mimics, miR-21 inhibitors, and control oligonucleotides were ordered from GenePharma (Shanghai, China). Sequences are as follows: miR-21 mimics, 5′-CAACAGCAGUCUGUAAGCUGGC-3′ (sense) and 5′-CAGCUUACAGACUGCUGUUGUU-3′ (antisense); negative control mimics, 5′-UUCUCCGAACGUGUCACGUTT-3′ (sense) and 5′-ACGUGACACGUUCGGAGAATT-3′ (antisense); miR-21 inhibitors, 5′-GCCAGCUUACAGACUGCUGUUG-3′ (chemically modified by 2′-Ome) and negative control inhibitors, 5′-CAGUACUUUUGUGUAGUACAA-3′. Macrophages were transfected with 50 nM of each oligonucleotides using Lipofectaime 2000 ^TM^ (Invitrogen) according to the manufacturer′s protocols. At 48 h post-transfection, the cells were stimulated with ultrapure LPS (24).

### RNA Interference

The IRAK4-specific siRNA (si-IRAK4) were 5′- GCAUCAUGUGAGGAGGUUUTT-3′ (sense) and 5′- AAACCUCCUCACAUGAUGCTT-3′ (antisense). The scrambled control RNA sequences were 5′-UUCUCCGAACGUGUCACGUTT-3′ (sense) and 5′-ACGUGACACGUUCGGAGAATT-3′ (antisense). Macrophages were transfected with each siRNA by using Lipofectamine 2000^TM^ for up to 48 h, and then stimulated with LPS for 3 and 6 h (24).

### RNA Extract and Quantitative Real-Time PCR

Total RNA was isolated with TRIzol Reagent (Invitrogen) according to the manufacturer's recommendation and the cDNA was synthesized using the FastQuant RT Kit (Tiangen) which includes DNase treatment of RNA to eliminate genomic contamination. The expression patterns of each gene were performed by qPCR on a 7500 system (Applied Biosystems, USA) using SYBR® Premix Ex Taq™ (Takara) (22, 28).

The small RNA was extracted by using miRcute miRNA Isolation Kit (Tiangen), and miRcute miRNA FirstStrand cDNA Synthesis Kit (Tiangen) was applied to reverse transcription of miRNAs. The expression analysis of miR-21 was executed by using the miRcute miRNA qPCR Detection Kit (Tiangen), following conditions: 94°C for 2 min, 40 cycles of two steps (94°C for 20 s, 60°C for 30 s, 72°C for 30 s). Quantification of the relative expression were done using the 2^−ΔΔ*CT*^ method after normalization to β-actin or 5.8s rRNA (29, 30). Three independent experiments were conducted for statistical analysis.

### Dual-Luciferase Reporter Assays

For miRNA target verification, the wild-type or mutant-type *IRAK4* 3′UTR luciferase reporters were cotransfected with miR-21 mimics, inhibitors, the pre-miR-21 plasmid, or negative controls into HEK293 cells. Reporter luciferase activities were measured using the Dual-Luciferase reporter assay system (Promega) (24). To determine the functional regulation of miR-21, HEK293 cells were cotransfected with NF-κB, IL-1β, and IL-8 luciferase reporter gene plasmids, *IRAK4* expression plasmid, pRL-TK Renilla luciferase plasmid, together with either miR-21 mimics, the pre-miR-21 plasmid or negative controls for dual-luciferase reporter assays (24). Afterwards, the cells were lysed for reporter activity testing using the Dual-Luciferase reporter assay system (Promega). All the luciferase activity values were achieved against the renilla luciferase control. For each experiment, three independent experiments were conducted, and each experiment was done in triplicate.

### Western Blotting

Cellular lysates were generated by using 1 × SDS-PAGE loading buffer. Proteins were extracted from cells and measured with the BCA Protein Assay kit (Vazyme), then subjected to SDS-PAGE (10%) gel and transferred to PVDF (Millipore) membranes by semidry blotting (Bio-Rad Trans Blot Turbo System) (24). The membranes were blocked with 5% BSA. Protein was blotted with different antibodies. The antibody against IRAK4 was diluted at 1:500 (Cell Signaling Technology), anti-Flag and anti-GAPDH monoclonal antibody were diluted at 1:2,000 (Sigma-Aldrich), and HRP-conjugated anti-rabbit IgG or anti-mouse IgG (Abbkine) at 1:5,000 (24). The results were the representative of three independent experiments. The immunoreactive proteins were detected by using WesternBright^TM^ ECL (Advansta). The digital imaging was performed with a cold CCD camera (24).

### Statistical Analysis

All the experiments were performed with at least three independent experiments, with three technical replicates for each experiment. The relative gene expression data was acquired using the 2^−ΔΔ*CT*^ method, and comparisons between groups were analyzed by one-way analysis of variance (ANOVA) followed by Duncan's multiple comparison tests (24, 29). Results are expressed as mean ± SE (standard error), and differences between means were considered to be statistically significant at *p-*values of < 0.05.

## Results

### LPS and *V. anguillarum* Enhance miR-21 Expression

**Figure 1:**
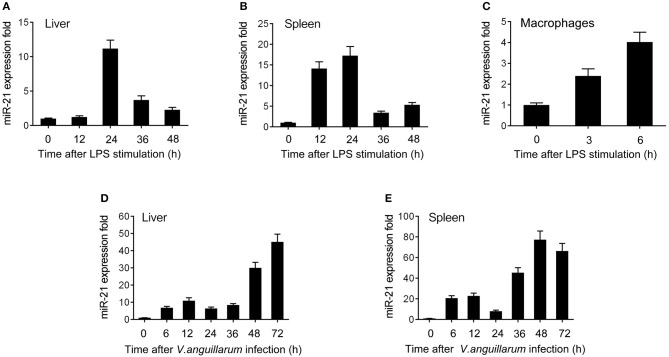
The expression profiles of miR-21 detected by qRT-PCR. Expression profiles of miR-21 in LPS stimulated miiuy croaker liver **(A)**, spleen **(B)**, and macrophages **(C)**. Expression profiles of miR-21 after *V. anguillarum* infection in liver **(D)** and spleen **(E)** samples. The data was normalized to 5.8S rRNA. Results are standardized to 1 in control samples. Data represented the mean ± SE from three independent triplicated experiments.

To explore the effect of Gram-negative bacteria on the miRNA profile, the miRNA profile in LPS-stimulated miiuy croaker spleen tissue was performed. Deep-sequencing data revealed that a set of miRNAs was differentially expressed upon LPS stimulation. Among these miRNAs, miR-21 was found to be significantly upregulated. Further validation of the miR-21 profile upon LPS stimulation was tested *in vivo* and *in vitro* using qRT-PCR. The results shown in Figures 1A,B revealed that miR-21 expression was significantly upregulated in LPS-stimulated liver and spleen, and it was expressed at the highest level in both liver and spleen at 24 h post-stimulation. Furthermore, the miR-21 profile was also investigated in macrophages, and the expression of miR-21 presented a significant increase after LPS stimulation (Figure 1C; Supplementary Figure 1). We also validated the expression of miR-21 under infection with the Gram-negative bacterium *V. anguillarum*. The results were similar to those observed upon LPS stimulation. As shown in Figures 1D,E; Supplementary Figure 1, the expression of miR-21 was highly expressed in liver and spleen samples, and reached its peak at 72 h and 48 h after infections, respectively. Collectively, these data indicated that miR-21 expression can be increased by LPS and *V. anguillarum* and that miR-21 may participate in the regulation of immune responses upon Gram-negative bacterial infection.

### miR-21 Represses the Expression Levels of LPS-Induced Inflammatory Cytokines

**Figure 2:**
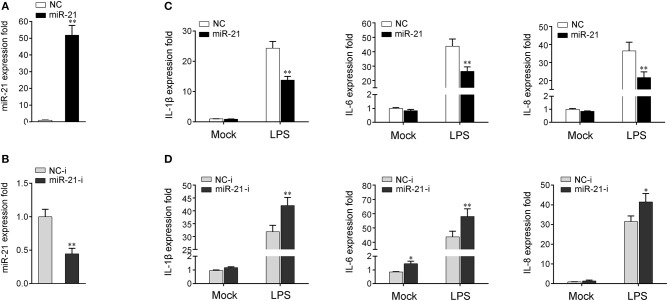
miR-21 suppresses LPS-induced inflammatory cytokine expression. **(A,B)** Miiuy croaker macrophages were transfected with control mimics (NC) or miR-21 mimics (miR-21) **(A)** and miR-21 inhibitors (miR-21-i) and control inhibitors (NC-i) **(B)** within a final concentration of 50 nM. At 48 h post-transfection, miR-21 expression was measured by qRT-PCR and normalized to 5.8S rRNA. **(C,D)** Macrophages were transfected with miR-21 or NC **(C)** and miR-21-i or NC-i **(D)** for 48 h. Then, the cells were stimulated with LPS for 6 h and the expression levels of IL-1β, IL-6, and IL-8 were analyzed by qRT-PCR. Results are standardized to 1 in control cells. Data are presented as the means ± SE from three independent triplicated experiments. **p < 0.01; *p < 0.05 vs. the controls.

To investigate whether miR-21 is involved in the regulation of the immune response during bacterial infection, its effect on the regulation of inflammatory cytokine expression was assessed after LPS stimulation. First, the effect of synthetic miR-21 mimics and inhibitors on miR-21 expression was evaluated in miiuy croaker macrophages. miRNA mimics are synthetic dsRNAs that simulate naturally occurring mature miRNAs, whereas miRNA inhibitors are chemically modified antisense ssRNAs that sequester and inhibit intracellular miRNAs (24). Miiuy croaker macrophages were transfected with miR-21 mimics or control mimics and miR-21 inhibitors or control inhibitors. As expected, transfection of miR-21 mimics significantly increased the miR-21 expression level in macrophages, whereas miR-21 inhibitors reduced its level (Figures 2A,B and Supplementary Figure 2). Next, the effects of miR-21 mimics and inhibitors on the expression levels of inflammatory cytokines were investigated in LPS-stimulated macrophages. We transfected with miR-21 mimics or control mimics into macrophages, and then challenged the cells with LPS for 6 h. As shown in Figure 2C and Supplementary Figure 2, the results from qRT-PCR analysis showed that transfection of miR-21 mimics inhibited the expression levels of LPS-induced IL-1β, IL-6, and IL-8. In contrast, as shown in Figure 2D and Supplementary Figure 2, the inhibition of endogenous miR-21 significantly increased inflammatory cytokines expression levels, including IL-1β, IL-6, and IL-8 compared with transfection of control inhibitors. Taken together, these data strongly demonstrated that miR-21 negatively regulates the expression of inflammatory cytokines in response to LPS stimulation, which indicated that miR-21 acts as a negative regulator involved in the immune response.

### IRAK4 Is a Target of miR-21

**Figure 3:**
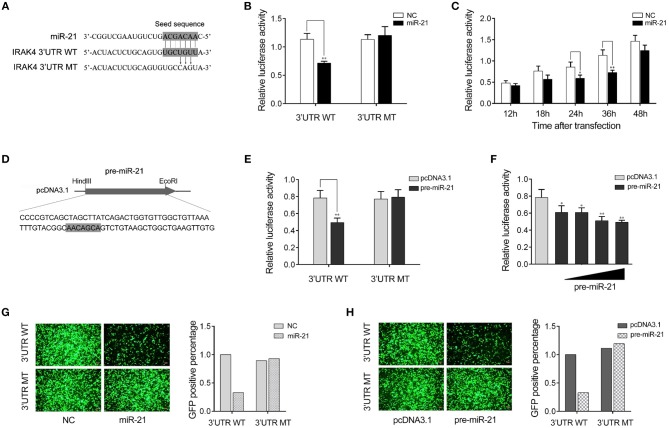
miR-21 targets miiuy croaker *IRAK4* gene. **(A)** Sequence alignment of miR-21 and its binding sites in the 3′ UTR of *IRAK4*. **(B)** HEK293 cells were transfected the wild-type *IRAK4* 3′UTR (WT) or the mutant *IRAK4* 3′UTR (MT), together with miR-21 mimics (miR-21) or control mimics (NC). The luciferase activity were measured using the Dual-Luciferase reporter assay system and normalized by Renilla luciferase activity. **(C)** The time gradient experiments was conducted for transfection. **(D)** The pre-miR-21 sequence and the process for its plasmid construction. **(E)** HEK293 cells were transfected the wild-type *IRAK4* 3′UTR (WT) or the mutant *IRAK4* 3′UTR (MT), together with the pre-miR-21 plasmid or pcDNA3.1 vector. The luciferase activity were measured and normalized by Renilla luciferase activity. **(F)** The concentration gradient experiments was conducted for transfection. **(G,H)** HEK293 cells were cotransfected with mVenus- *IRAK4*-3′UTR (3′UTR WT) or the mutant type (3′UTR MT), together with miR-21 or NC **(G)** and the pre-miR-21 or pcDNA3.1 **(H)**. At 48 h post-transfection, the fluorescence intensity was evaluated. Data are presented as the means ± SE from at least three independent triplicated experiments. **p < 0.01; *p < 0.05 vs. the controls.

We next verify the potential target gene of miR-21. The bioinformatics software programs were used to search for miR-21 targets, and we found a putative miR-21 binding site in the 3′UTR of *IRAK4* gene (Figure 3A). To obtain direct evidence that *IRAK4* is a target of miR-21, luciferase reporter constructs were generated by cloning either the wild-type 3′UTR or the mutant 3′UTR of miiuy croaker *IRAK4* into pmir-GLO vector, and the mutant IRKA4 3′UTR luciferase reporter plasmid contains the miR-21 target sequence mutations in the seed region (Figure 3A). After cotransfection of luciferase reporter plasmids and miR-21 mimics or control mimics into HEK293 cells, we observed that miR-21 mimics markedly inhibited the luciferase activity of cells when the wild-type 3′UTR was transfected, whereas, miR-21 mimics showed no effect on the luciferase activity of cells transfected with the mutant of *IRAK4* 3′UTR (Figure 3B). Furthermore, the gradient experiments of transfection time were conducted with the miR-21 mimics. As shown in Figure 3C, the results indicated that miR-21 mimics can inhibit the luciferase activity within 18 h to 48 h after transfection, and it significantly inhibited the luciferase activity at 36 h after transfection.

Given that miRNA processing system is conserved from invertebrates to vertebrates (31), we constructed the pre-miR-21 sequence plasmid and then transfected it into HEK293 cells for *in vitro* expression (Figure 3D). After transfection of the pre-miR-21 plasmid into HEK293 cells, we observed that overexpression of pre-miR-21 could also decrease luciferase activity, whereas no change of luciferase activity was observed in cells transfected with the mutant IRKA4 3′UTR (Figure 3E). To further verify the results, the concentration gradient experiments for the pre-miR-21 were conducted. As shown in Figure 3F, the results indicated that pre-miR-21 could reduce the level of the luciferase activity in a dose-dependent manner. Additionally, the miR-21 mimics and the pre-miR-21 plasmid could also downregulate green fluorescent protein (GFP) gene expression when the *IRAK4* 3′UTR was cloned into the 3′UTR region of the GFP vector, whereas no change in fluorescence intensity was observed in cells transfected with the mutant *IRAK4* 3′UTR (Figures 3G,H). Above all, these data adequately demonstrated that the nucleotide sequence in the 3′UTR of *IRAK4* is a potential miR-21 targeting site.

### miR-21 Regulates IRAK4 Expression by Inhibition Protein Translation but Not Degradation mRNA

**Figure 4:**
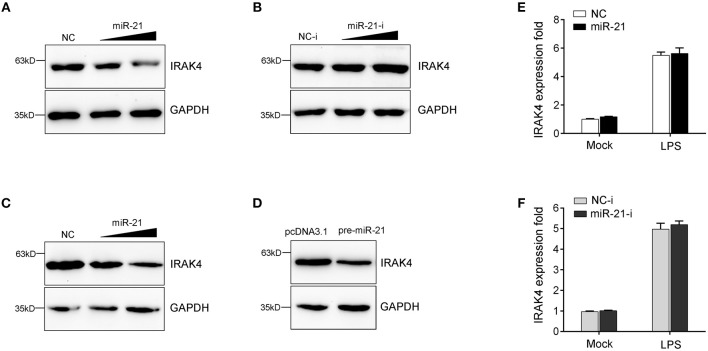
miR-21 suppresses the protein levels of IRAK4 expression. **(A)** Miiuy croaker macrophages were transfected with miR-21 (0, 30, and 60 nM) or control mimics (60, 30, and 0 nM), and the protein levels of IRAK4 were determined by Western blot. **(B)** Macrophages were transfected with miR-21 inhibitors (0, 30, and 60 nM) or control inhibitors (60, 30, and 0 nM), the protein levels of IRAK4 were determined by Western blot. **(C)** HEK293 cells were cotransfected with IRAK4 expression plasmid, along with miR-21 (0, 30, and 60 nM) or control mimics (60, 30, and 0 nM), and the protein levels of IRAK4 were determined by Western blot. **(D)** HEK293 cells were cotransfected with IRAK4 expression plasmid, along with the pre-miR-21 plasmid or pcDNA 3.1 vector. At 48 h post-transfection, the protein levels of IRAK4 were determined by Western blot. **(E,F)** The miiuy croaker macrophages were transfected with miR-21 or control mimics **(E)** and miR-21 inhibitors or control inhibitors **(F)** for 48 h and then stimulated with LPS. The expression level of IRAK4 were measured by real-time qPCR and normalized to β-actin. Data are presented as the means ± SE from three independent triplicated experiments.

MicroRNAs function mainly by binding to the 3′UTR of target mRNA to achieve posttranscriptional regulation of gene expression, resulting in protein translation repression or mRNA degradation. To examine miR-21 function in the regulation of IRAK4 expression, we transfected with miR-21 mimics or inhibitors into miiuy croaker macrophages and measured the expression of endogenous IRAK4. As shown in Figure 4A, transfection of miR-21 mimics significantly decreased the protein level of IRAK4, and the inhibition effect was shown in a dose-dependent manner. On the contrary, as shown in Figure 4B, the miR-21 inhibitors obviously increased the protein level of IRAK4 in a dose-dependent manner. The results suggested that miR-21 could negatively regulate the protein level of endogenous IRAK4. To further examine the regulation mechanism of miR-21, an IRAK4 expression plasmid was constructed and cotransfected into HEK293 cells. To construct the IRAK4 expression plasmid, we first amplified the full-length coding DNA sequence (CDS) region and 3′UTR of the miiuy croaker *IRAK4* gene, and cloned the sequence into the pcDNA3.1 vector. Then, the IRAK4 expression plasmids were cotransfected with the miR-21 mimics, the pre-miR-21 plasmid or their controls into HEK293 cells. As expected, the results showed that miR-21 mimics could significantly suppress the protein expression of IRAK4 in a dose-dependent manner (Figure 4C). A consistent effect also produced by the pre-miR-21 plasmid (Figure 4D). These results indicated that miR-21 can inhibit the expression of IRAK4 in a manner that inhibits translation. Additionally, we further investigated whether miR-21 also acts to affect the stability of IRAK4 mRNA. To this end, macrophages were transfected with miR-21 mimics or inhibitors, and then treated with LPS. As shown in Figures 4E,F, neither transfection of miR-21 mimics nor miR-21 inhibitors has any effect on the mRNA levels of IRAK4, which indicate that miR-21 could not affect the stability of IRAK4 mRNA. Collectively, these results suggested that IRAK4 is a direct target of miR-21, and the induction of miR-21 decreases IRAK4 protein expression by repressing its protein translation but not by affecting its mRNA stability.

### miR-21 Suppresses IRAK4-Mediated NF-κB Signaling

**Figure 5:**
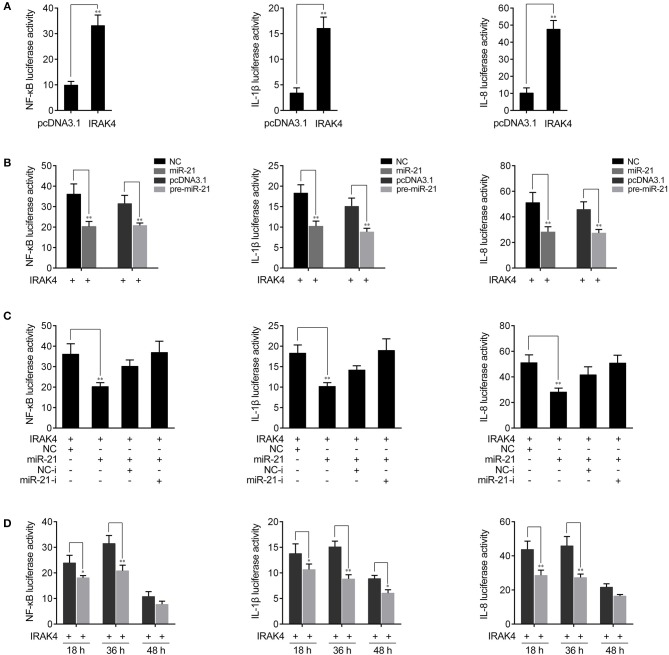
miR-21 inhibits NF-κB signaling. **(A)** HEK293 cells were cotransfected with IRAK4 expression plasmid or pcDNA3.1 vector, together with pRL-TK Renilla luciferase plasmid, luciferase reporter genes NF-κB, IL-1β, or IL-8. The luciferase activity was measured and pcDNA3.1 empty vector were used to control the same amount of molecules for transfections. **(B)** HEK293 cells were cotransfected with miR-21 mimics, control mimics, the pre-miR-21 plasmid or pcDNA3.1 vector, together with NF-κB, IL-1β, or IL-8 luciferase reporters and IRAK4 expression plasmid. Control mimics and pcDNA3.1 vector were used to control the same amount of molecules for transfections. **(C)** HEK293 cells were transfected with miR-21 mimics, control mimics, miR-21 inhibitors or control inhibitors, together with NF-κB, IL-1β, or IL-8 luciferase reporters, IRAK4 expression plasmid. For each transfections, the total amount of oligonucleotides were controlled and normalized (final concentration, 50 nM). **(D)** The time gradient experiments was conducted for transfection. Luciferase activity was normalized to renilla luciferase activity. All data are representative of at least three independent experiments. **p < 0.01; *p < 0.05 vs. the controls.

Previous studies reported that the activation of NF-κB signaling promotes the expression of various inflammatory genes (7, 8). Given that miR-21 regulates the expression of inflammatory cytokines in miiuy croaker, we thus want to examine whether miR-21 affects inflammatory cytokine expression by the activation of NF-κB signaling pathway. To this end, we first measured whether its target gene *IRAK4* is involved in regulating NF-κB signaling. A series of assays was performed to monitor the activations of NF-κB, IL-1β, and IL-8 reporter genes after the IRAK4 expression plasmid was transfected into HEK293 cells. As shown in Figure 5A, the results showed that the overexpression of miiuy croaker IRAK4 efficiently activated the NF-κB reporter gene as well as IL-1β and IL-8 reporter genes. Given that miR-21 targets *IRAK4* and negatively regulates its expression, we next examined whether miR-21 could regulate NF-κB, IL-1β, and IL-8 signaling. We transfected with the IRAK4 expression plasmid, together with miR-21 mimics, the pre-miR-21 plasmid or their controls into HEK293 cells. As shown in Figure 5B, miR-21 mimics, as well as the pre-miR-21 plasmid, could markedly suppress the activation of NF-κB, IL-1β, and IL-8 induced by overexpression of IRAK4, compared with negative control groups. We further investigated the down-regulation mechanism by cotransfection of miR-21 mimics and inhibitors. As shown in Figure 5C, the inhibition effect produced by miR-21 mimics was attenuated after cotransfection with miR-21 inhibitors. Furthermore, the gradient experiments of transfection time were conducted by the pre-miR-21 plasmid. As shown in Figure 5D, the results indicated that pre-miR-21 can suppress the activation of NF-κB, IL-1β, and IL-8 report genes within 18 h to 48 h after transfection, and it significantly inhibited the luciferase activity at 36 h post-transfection. Taken together, these results indicated that miR-21 is able to suppress IRAK4-mediated NF-κB signaling pathway and negatively regulates the activation of NF-κB, IL-1β, and IL-8 signaling.

### Knockdown of IRAK4 Inhibits Inflammatory Cytokine Expression

**Figure 6:**
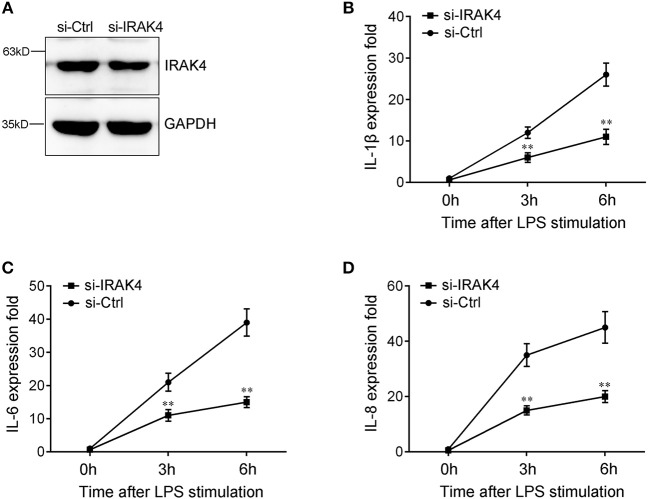
The expression levels of inflammatory cytokines after IRAK4 interference. **(A)** Miiuy croaker macrophages were transfected with control siRNA (si-Ctrl) or IRAK4 siRNA (si-IRAK4). After 48 h, IRAK4 protein levels were determined by Western blot. **(B–D)** After 48 h transfected with si-Ctrl or si-IRAK4, macrophages were then stimulated with LPS for 3 h or 6 h. The expression levels IL-1β **(B)**, IL-6 **(C)**, and IL-8 **(D)** were determined and normalized to β-actin. All data are representative of at least three independent experiments. **p < 0.01 vs. the controls.

To confirm the contribution of IRAK4 to the inflammatory response, we silenced IRAK4 and examined the expression of inflammatory cytokines upon LPS stimulation. As shown in Figure 6A, IRAK4-specific siRNA effectively inhibited IRAK4 expression in miiuy croaker macrophages (32). Knockdown of IRAK4 significantly decreased the expression of IL-1β in macrophages, which produced an effect similar to that of miR-21 overexpression (Figure 6B). Similar downregulation trends were also detected for IL-6 and IL-8 (Figures 6C,D). These results indicated that miR-21 regulates inflammatory response through suppression of endogenous IRAK4, thereby inhibiting inflammatory cytokine expression.

### miR-21-Regulating IRAK4 Gene Has Been Found in Other Teleost

**Figure 7:**
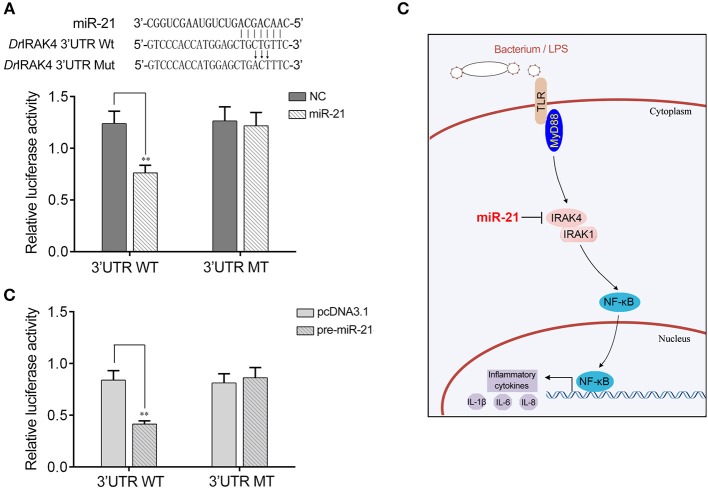
A proposed model for the mechanism regulated by miR-21 in fish species. **(A)** Schematic diagram of the predicted target sites of miR-21 in 3′UTR of *Danio rerio* IRAK4. HEK293 cells were transfected with miR-21 mimics or control mimics, along with the wild type of *D. rerio IRAK4* 3′UTR (WT) or the mutant *D. rerio IRAK4*-3′UTR (MT) for 24 h and the luciferase activity was determined. **(B)** HEK293 cells were transfected with the pre-miR-21 plasmid or pcDNA3.1, together with the wild type of *D. rerio IRAK4* 3′UTR (WT) or the mutant *D. rerio IRAK4*-3′UTR (MT). After 36 h post-transfection, the luciferase activity was determined. **(C)** A proposed model for the mechanism by which induced miR-21 negatively regulates inflammatory cytokines production in a manner by targeting *IRAK4* and inhibiting NF-κB signaling, thereby inhibiting inflammatory response.

To address the generality of our findings that miR-21 targets and regulates *IRAK4*, we explored the results in other model animals. To this end, luciferase reporter constructs were generated by cloning zebrafish *IRAK4* 3′UTR into pmir-GLO vector within the mutation at the miR-21 binding site as a control (Figure 7A). As shown in the dual-luciferase reporter assays, miR-21 mimics were sufficient to decrease the luciferase activity when cotransfected with the zebrafish *IRAK4* 3′UTR reporter plasmid into HEK293 cells, whereas miR-21 mimics showed no effect on the luciferase activity of cells transfected with a mutant-type (Figure 7A). We also found that pre-miR-21 plasmid presented a similar effect on the inhibition of luciferase activities (Figure 7B). These data indicated that miR-21 could also target zebrafish *IRAK4* 3′UTR, which will provide other fish species with the mechanism to regulate of inflammatory responses. Taken together, these results indicate that a model could be proposed in which miR-21 inhibits inflammatory cytokine production by targeting fish *IRAK4* and suppressing NF-κB signaling, thereby avoiding excessive inflammation (Figure 7C).

## Discussion

The innate immune and inflammatory responses play crucial roles in eliminating pathogens and resisting pathogen invasion. The complex process of inflammatory response is triggered by innate immune system receptors, which recognize pathogens and precisely regulate immune response (6). Excessive immune response and inflammation will disrupt immune homeostasis and further induce autoimmune and inflammatory diseases. Hence, various layers of negative mechanisms and distinct molecules participate in controlling the homeostasis of immune system and avoiding excessive inflammation. As the complexity of the living environment, fish are constantly threatened by pathogenic microorganisms. In fish, diseases caused by bacterial infections have caused high mortality and severe economic losses because of their wide prevalence and high incidence. Thus, understanding how to regulate the immune response and inflammation during bacterial infections will facilitate the prevention and treatment of bacterial diseases. In the present study, we demonstrated that miR-21 acts as negative effectors involved in the regulation of fish inflammatory response upon Gram-negative bacterial infections. Specifically, we found that *V. anguillarum* and LPS significantly increases the expression of miR-21 in miiuy croaker. Upregulated miR-21 represses the production of LPS-induced inflammatory cytokines through targeting *IRAK4* and negative regulating NF-κB signaling, thereby avoiding excessive inflammation. These findings indicated that miR-21 acts as “fine-tuners” involved in the fish immune response against infections, which throws light into the role of miRNAs in the regulation of host-pathogen interaction networks.

TLRs that arguably represent the best-studied pathogen-detection sensors have a central role in linking pathogen recognition to the induction of activation signaling pathways and inducing the expression of immune and pro-inflammatory genes. To achieve the balanced output and prevent inappropriate activation of TLRs, the TLR-signaling pathways are strictly and finely regulated by a series of molecules (33, 34). For example, MyD88s, the MyD88 antagonist, has been identified as an inhibitor of TLR signaling through interaction with TLR9 (34). The LPS-induced kinase inactivating form of IRAKM plays as a negative regulator by inhibiting phosphorylation of IRAK1 (35). The LPS and CpG DNA induced SOCS1 also participates in regulating TLR signaling by inhibiting IRAKs' functions in macrophages (36).

Recently, miRNAs have received considerable attention as the newly identified family of regulators involved in fine-tuning the TLR-signaling pathways. In mammals, miRNAs, such as let-7e, let-7i, miR-125b, miR-146, miR-155, and miR-223, have been shown to act as negative regulators involved in TLR-signaling pathways (12, 37). However, due to the limitations of research materials and methods in fish, the role of miRNAs in immune responses are just beginning to be studied. In miiuy croaker, several miRNAs have recently been shown to target TLR signaling proteins and transcriptional factors. MyD88, the crucial adaptor protein, has been identified to be regulated by miR-3570 which eventually attenuates inflammatory cytokine production (38). miR-203 regulates TLR signaling by decreasing IRAK4 expression (28). miR-216a directly targets p65, a key component of the NF-κB family, thereby acting as a negative regulator of inflammation in miiuy croaker (24). In addition, a single molecule in the TLR pathway can be targeted by multiple miRNAs in mammals. For example, mammal miR-16, miR-125b, miR-155, miR-221, and miR-579 can target TNF-α (18, 36). In fish, a previous study has reported that *IRAK4* can be targeted by miR-203. Herein, we found another miRNA, miR-21, which acts as a negative regulator in inflammation by directly targeting *IRAK4*. These results indicated that fish miRNA can also target several distinct mRNAs. Collectively, these findings in miiuy croaker expand the knowledge of the immune regulatory networks of miRNA in fish, which also enriched our knowledge of the intricate networks of host-pathogen interaction.

Upon effective recognition, PRRs subsequent stimulate nuclear factor NF-κB signaling to activate innate and adaptive immunity aiming at eliminating detected pathogens and developing a lasting protection against future infections. NF-κB is an important transcription factor that regulates the transcription and expression of a large set of genes. As reported, miRNAs play crucial roles in regulating the NF-κB signaling network. Certain miRNAs contribute to the activation of NF-κB, for example, miR-301a and miR-381 are identified miRNAs that activate NF-κB (39, 40). Conversely, many miRNAs are involved in negatively regulating NF-κB signaling. For example, miR-146a suppresses NF-κB activity by inhibiting the expression of TRAF6 and IRAK1 (41); miR-7578 identified in epididymis could regulate the inflammatory response through inhibiting NF-κB signaling (42). These studies form a miRNA network in regulating NF-κB signaling for mammals, whereas the underlying mechanisms of miRNAs in fish are just beginning to be explored. In this study, endogenous miR-21 in miiuy croaker was found to negatively regulate NF-κB signaling. One finding is that the miR-21-mediated regulation mechanism occurs by targeting *IRAK4*.

IRAK4 is a member of the IL-1 receptor-associated kinase (IRAK) family, which have key roles in regulating of inflammatory responses. Upon binding of a TLR/MyD88 complex, IRAK4 functions as a kinase that acts upstream of IRAK and phosphorylates IRAK. Phosphorylated IRAK then mediates the recruitment of TRAF6 to the receptor complex (43). Dissociation from the receptor complex, IRAK-TRAF6 complex interacts with and phosphorylates TAK1. Subsequently, phosphorylation of TAK1 leads to the activation of NF-κB and the transcription of proinflammatory cytokines (44). Hence, IRAK4 is the indispensable pivotal adaptor kinase in almost all TLR signaling. MicroRNA-21 has been shown to promote the cell proliferation, invasion, and migration abilities in ovarian epithelial carcinomas through targeting PTEN (45). Moreover, many studies suggest that miR-21 functions as an oncomiR that is up-regulated in many types of cancers, which has brought it to the forefront of cancer research (46). However, very little is known regarding the role of miR-21 in inflammatory reaction. In this study, miR-21 has been validated as a negative regulator involved in controlling excessive inflammation by targeting *IRAK4*. The results provide useful insight into the role of miR-21 in the regulation of immune and inflammatory responses.

In summary, this study demonstrated that miR-21 acts as a negative regulator in the inflammatory response to Gram-negative bacteria infections. The underlying regulatory mechanism is through directly targeting *IRAK4* and subsequently repressing NF-κB signaling. These findings suggest the essential role of miR-21 in the inhibition of an excessive inflammation reaction and also provide a new miRNA-mediated regulation mechanism against pathogen.

## Author Contributions

TX conceived and designed the experiments. QC, XY, and LL performed the experiments. QC analyzed the data. QC and TX wrote the paper.

### Conflict of Interest Statement

The authors declare that the research was conducted in the absence of any commercial or financial relationships that could be construed as a potential conflict of interest. The reviewer PC and handling editor declared their shared affiliation.
