# Supplementary material for: The Inducible microRNA-21 Negatively Modulates the Inflammatory Response in Teleost Fish via Targeting IRAK4
Source: Front Immunol. 2019 Jul 16;10:1623. doi: 10.3389/fimmu.2019.01623 (PMC6648887; doi:10.3389/fimmu.2019.01623)
Supplement: Supplementary file 1 [file Data_Sheet_1.PDF]

Supplementary material

The inducible microRNA-21 negatively modulates the inflammatory response in teleost fish via targeting IRAK4

Qing Chu[1,3,4], Xiaolong Yan[1,3,4], Lihua Liu[1,3,4], Tianjun Xu[1,2,3,4,5*]

1. *Laboratory of Fish Molecular Immunology, College of Fisheries and Life Science, Shanghai Ocean University, Shanghai, 201306, China*
2. *Laboratory of Marine Biology and Biotechnology, Qingdao National Laboratory for Marine Science and Technology, Qingdao, China*
3. *National Pathogen Collection Center for Aquatic Animals, Shanghai Ocean University, 201306, China*
4. *Key Laboratory of Exploration and Utilization of Aquatic Genetic Resources (Shanghai Ocean University), Ministry of Education, 201306, China*
5. *International Research Center for Marine Biosciences at Shanghai Ocean University, Ministry of Science and Technology, 201306, China*

*Corresponding author. Dr. Tianjun Xu

E-mail: tianjunxu@163. com

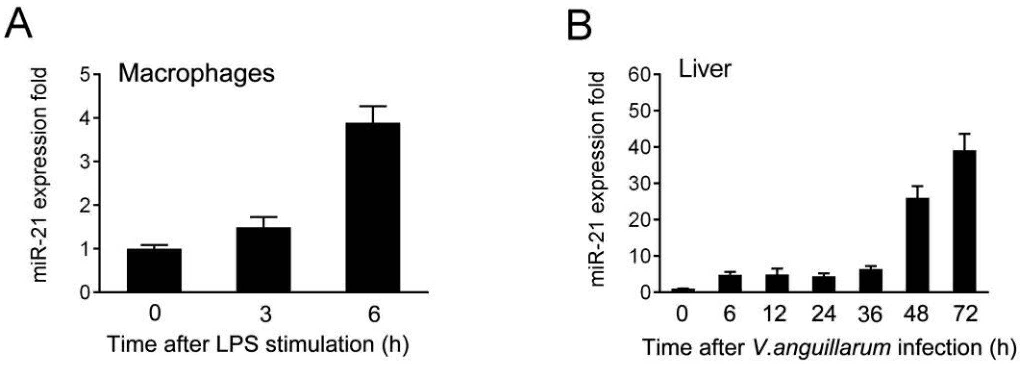

Supplementary Figure 1. The supplementary experiment for the expression analysis of miR-21. (A) Expression profiles of miR-21 in LPS stimulated miiuy croaker macrophages. (B) Expression profiles of miR-21 after *V. anguillarum* infection in liver. The relative expression of miRNA were normalized to that of internal control U6 snRNA expression. Results are standardized to 1 in control samples. Data represented the mean ± SE from three independent triplicated experiments.

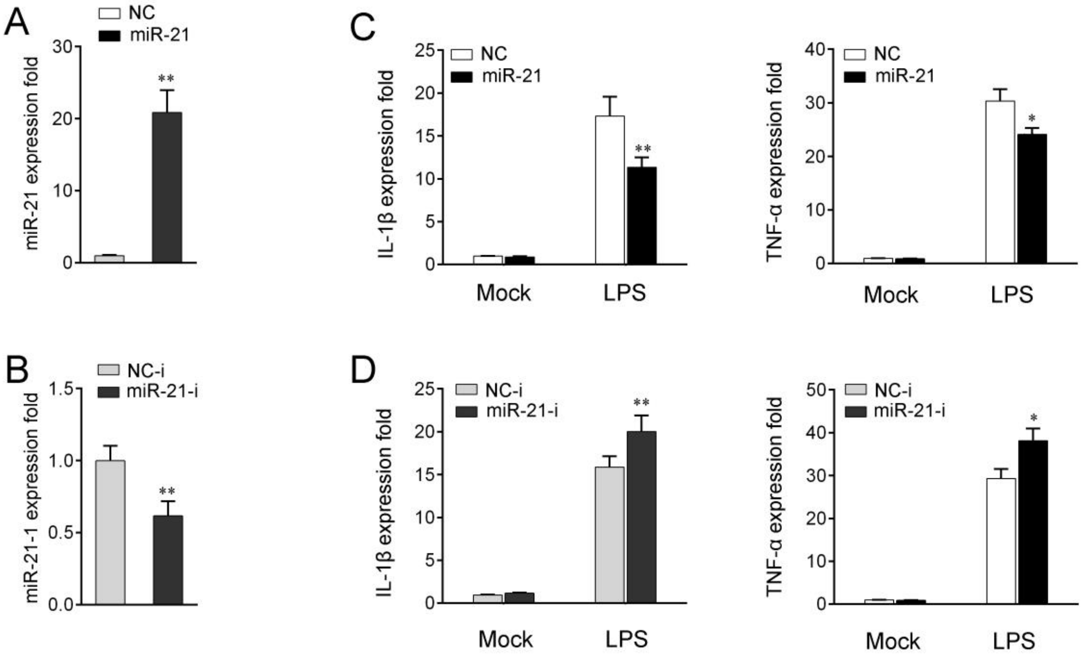

Supplementary Figure 2. The supplementary experiment for miR-21 suppresses LPS-induced inflammatory cytokine expression. (A, B) Miiuy croaker macrophages were transfected with control mimics (NC) or miR-21 mimics (miR-21) (A) and miR-21 inhibitors (miR-21-i) and control inhibitors (NC-i) (B). At 48 h post-transfection, miR-21 expression was measured by qRT-PCR and normalized by U6 snRNA expression. (C, D) Macrophages were transfected with miR-21 or NC (C) and miR-21-i or NC-i (D) for 48 h. Then, the cells were stimulated with LPS and the expression levels of IL-1β and TNF-α were analyzed by qRT-PCR and normalized by glyceraldehyde 3-phosphate dehydrogenase (GAPDH) expression. Results are standardized to 1 in control cells. Data are presented as the means ± SE from three independent triplicated experiments. **, *$p < 0.01$*; *, *$p < 0.05$* versus the controls.
